# Prevalence and distribution of primary glomerular diseases in Africa: a systematic review and meta-analysis of observational studies

**DOI:** 10.11604/pamj.2023.45.153.40741

**Published:** 2023-08-09

**Authors:** Udeme Ekpenyong Ekrikpo, Patience Ngozi Obiagwu, Aniema Isaac Udo, Ijezie Innocent Chukwuonye, Jean Jacques Noubiap, Ugochi Sophia Okpechi-Samuel, Udeme-Abasi Nelson Udoudo, Elliot Koranteng Tannor, Stanley Chidozie Ngoka, Ikechukwu Okeke Mbah, Aminu Kasarawa Bello, Ikechi Gareth Okpechi

**Affiliations:** 1 Department of Medicine, University of Uyo, Uyo, Nigeria,; 2 Department of Paediatrics, Bayero University, Aminu Kano Teaching Hospital, Kano, Nigeria,; 3 Department of Internal Medicine, Federal Medical Centre, Umuahia, Nigeria,; 4 Division of Cardiology, Department of Medicine, University of California-San Francisco, San Francisco, California, USA,; 5 Department of Internal Medicine, Federal Medical Centre, Abuja, Nigeria,; 6 Department of Medicine, Kwame Nkrumah University of Science and Technology, Kumasi, Ghana,; 7 Renal Unit, Department of Medicine, Komfo Anokye Teaching Hospital, Kumasi, Ghana,; 8 Department of Internal Medicine, Federal University Teaching Hospital, Owerri, Nigeria,; 9 Division of Nephrology, Bingham University Teaching Hospital, Jos, Nigeria,; 10 Division of Nephrology, Department of Medicine, University of Alberta, Edmonton, Canada

**Keywords:** Glomerulonephritis, kidney biopsy, minimal change disease, focal segmental glomerulosclerosis, IgA nephropathy, Africa

## Abstract

Glomerulonephritis (GN) is a predominant cause of kidney failure in Africa. The prevalence of primary GNs varies widely across Africa depending on the relative proportion of secondary GNs and genetic predispositions. We assessed the overall and sub-regional prevalence of primary GN and its histologic subtypes in Africa. We searched PubMed, EMBASE and African Journals Online for studies of biopsy-proven primary GNs across all age groups in Africa published between 2010 and 2022. Data for primary GNs [minimal change disease (MCD), focal segmental glomerulosclerosis (FSGS), membranous nephropathy (MN), mesangioproliferative GN (MesPGN), membranoproliferative GN (MPGN), post-infectious GN (PIGN), IgA Nephropathy (IgAN), and crescentic GN (CresGN)] were extracted. Pooled prevalence was determined using the random effects model. Seventeen eligible articles (n = 6,494 individuals) from 8 African countries met the inclusion criteria. The overall pooled prevalence of FSGS, MCD, MN, MPGN, MesPGN, PIGN, IgAN and CresGN was 26.10%, 22.40%, 8.40%, 6.40%, 6.40%, 2.60%, 2.60%, 1.40%, respectively. Only 4 studies (23.5%) used light microscopy (LM), immunofluorescence (IF), and electron microscopy (EM) for diagnosis. There were significant differences in the distribution of histologic subtypes in the paediatric compared to the adult population and across geographic sub-regions, with West Africa having a higher prevalence of FSGS. Overall, the dominance of FSGS across most regions and age groups has implications for disease diagnosis and ongoing care. Research efforts to understand the impact of this trend on kidney disease outcomes and efforts to improve kidney biopsy practice as a means of early disease detection are needed in Africa.

## Introduction

Over the last three decades, glomerulonephritis has remained the predominant cause of chronic kidney disease (CKD) in Africa, with wide variation across the regions [1,2]. In North Africa, diabetes only recently surpassed glomerulonephritis as the most predominant CKD aetiology [3]. In sub-Saharan Africa (SSA), a region with 48 countries and a combined population of about 1.2 billion inhabitants [4] glomerulonephritis has persisted as the most common cause of CKD in incident kidney failure patients [5,6] The prevalence of glomerulonephritis as a cause of kidney failure varies across sub-Saharan Africa ranging from 8.1% to 36.1% in West Africa [7-11], 10% in East Africa [12], and 25% to 32% in South Africa [13]. A complex combination of bacterial, viral and parasitic infections coupled with a well-documented genetic predisposition, the Apolipoprotein L1 (*APOL1*) kidney risk variants have sustained the importance of glomerulonephritis as a cause of kidney failure in Africa [14].

Although IgA nephropathy (IgAN) has been identified as the most prevalent primary glomerular disease in many European and Asian countries [15-17], focal segmental glomerulosclerosis (FSGS) is the most common type in other countries with increased ethnic diversity [18]. A previous African study identified minimal change disease (MCD; 16.5%) and FSGS (15.9%) to be the most common primary glomerular disease in the continent and highlighted regional differences [19]. Paediatric studies in Africa have also shown changes in primary glomerular disease patterns in this population [20]. It is important to document possible changes in trends over time, especially of primary glomerulonephritis, given their importance to the high burden of kidney failure in Africa. Even though there are few kidney registries in Africa, documented changes identified in this study will be likely useful to improve diagnostic and treatment accuracy for clinicians and policy planning for health systems for kidney care in Africa [15]. We, therefore, conducted this systematic review and meta-analysis of studies reporting biopsy-proven primary glomerulonephritis across Africa to document the burden of PGN in Africa.

## Methods

This study was registered with the International Prospective Register of Systematic Reviews (PROSPERO) under number CRD42022349859 and was conducted in accordance with the updated Preferred Reporting Items for Systematic Reviews and Meta-Analyses (PRISMA) [21] framework.

**Figure 1:**
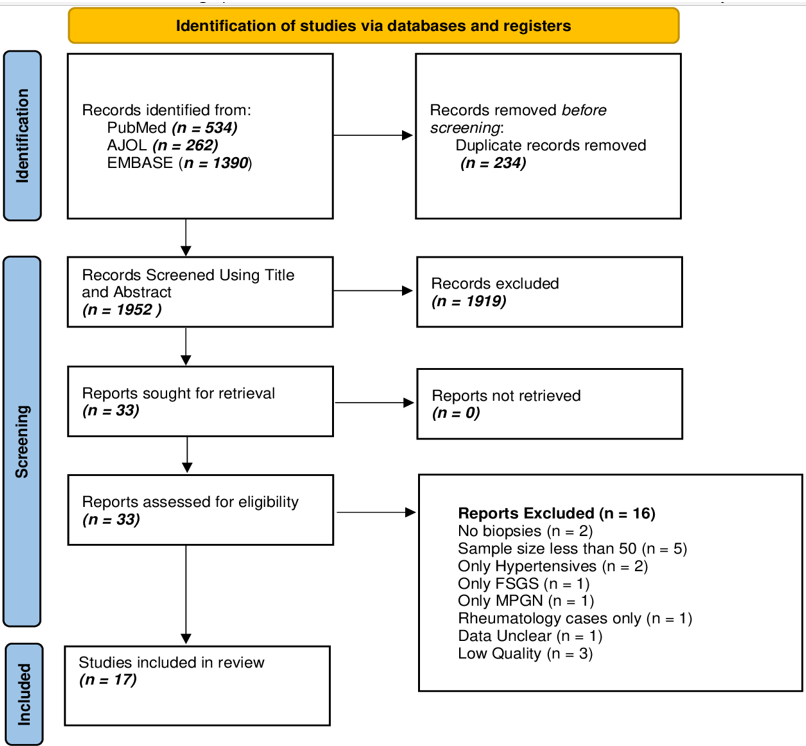
PRISMA flow chart of study selection

**Search strategy:** relevant studies were identified by searching PubMed, Excerpta Medica database (EMBASE), and African Journals Online (AJOL) (Figure 1). The search was limited to studies from African countries, published from January 1^st^ 2010 to June 30^th^ 2022, and without any language restriction. A further search of the bibliographies of the articles obtained from the database search was undertaken to identify other articles for inclusion in the study. The search strategy included only terms relating to or describing the spectrum of biopsy-proven primary glomerular diseases in Africa. This included 'glomerulonephritis', 'IgA nephropathy', 'membranous nephropathy', 'membranoproliferative glomerulonephritis', 'mesangial proliferative glomerulonephritis', 'minimal change disease', 'focal segmental glomerulosclerosis', 'post-infectious glomerulonephritis', 'idiopathic crescentic proliferative glomerulonephritis'. The search strategies for PubMed and EMBASE are presented in Annex 1.

**Data collection:** two authors (Udeme Ekpenyong Ekrikpo and Patience Ngozi Obiagwu) independently assessed titles and abstracts for eligibility. Disagreements between the two reviewers were resolved by a third reviewer (IGO). The inclusion criteria included: 1) adult and paediatric studies undertaken only in Africa; 2) studies reporting biopsy-proven cases of PGN diagnosed using light microscopy, immunofluorescence, and/or electron microscopy; 3) enrolled a minimum of 50 participants; 4) provided data on the demographic distribution and histopathologic types of glomerular disease sub-types. Data collected included: year of publication, country, and African sub-region, study sample size, clinical indication for biopsy, mean age at biopsy, gender distribution, and the proportion of all biopsies that constitute primary glomerular diseases. The relative proportions of MCD, FSGS, IgAN, membranous nephropathy (MN), membranoproliferative glomerulonephritis (MPGN), mesangioproliferative glomerulonephritis (MesPGN), post-infectious glomerulonephritis (PIGN) and primary crescentic glomerulonephritis (CGN) were also recorded. The quality of included studies was assessed using the Joanna-Briggs critical appraisal tool for prevalence studies [22]. Studies with a score of 0-3 were regarded as low quality, 4-6 as medium quality, and 7-9 as high-quality studies.

**Data extraction and synthesis:** data extraction was undertaken independently by UEE and PO, while data validation after extraction was done by AIA and UEE. The DerSimonian-Laird random effects models were used to determine the pooled prevalence of the different histologic sub-types of PGN after stabilization of the prevalence from the constituent studies using the Freeman-Tukey double arcsine transformation [23]. Heterogeneity across studies was assessed using the Cochran Q and I2 statistics [24], while Egger's [25] and Begg's [26] tests were used to assess publication bias. Sub-group analyses were also undertaken to compare prevalence and patterns across African sub-regions and age groups. Pooled percentages (95% confidence interval) were used to report primary glomerular disease sub-types, while the median (interquartile range [QR]) was used to report demographic features of included studies and indications of kidney biopsy. All analyses used Stata 17.0 (StataCorp, Texas, USA).

## Results

The initial literature search retrieved 1954 articles after the removal of duplicates, of which 33 were selected after the title and abstract screening for full-text review. We identified 17 articles [27-43], that were eligible and included in this systematic review (Figure 1). The included studies have a combined sample size of 6,494 individuals from 8 African countries (Egypt, Morocco, Sudan, Ivory Coast, Gambia, Senegal, Kenya, and South Africa). There were 1,354 participants (9 studies) from North Africa [27-30,32,33,37,39,43], 1,584 (4 studies) from West Africa [34,35,40,41], 193 (1 study) from East Africa [36], and 3,363 (3 studies) from Southern Africa [31,38,42]. Ten of the studies [29,32,34-38,40,42,43] included only adults, five reported data only in paediatrics [28,30,31,33,39], while two studies reported adult and paediatric data [27,41]. The average age of the patients ranged from 5.2 to 44.8 years. The median proportion of males across the studies was 53.5% (IQR 51.8 - 61.0%). Nephrotic syndrome (61.5% [IQR 47.7 - 100%]), abnormal kidney function (11.2% [IQR 0.0 - 16.6%]), and nephritic syndrome, 1.0% (IQR 0 - 6.7%) were the most prevalent indications for performing kidney biopsy. Eleven (64.7%) studies were of high quality [29-31,35-39,41-43], and 6 (35.3%) were of medium quality [27,28,32-34,40]. Method of histopathological assessment was not reported in 4 studies (23.5%) [33,34,40,44] 2 studies (11.8%) only used LM [28,36] 7 studies (41.2%) only used LM and IHC/IF [27,29,32,35,37,39,43], while 4 studies (23.5%) reported use of LM, IHC/IF, and EM [30,31,38,42] for reporting histologies. High-quality studies were from East Africa (n=1) [36], Southern Africa (n=3) [31,38,42], West Africa (n=2) [35,41] and North Africa (n=5) [29,30,37,39,43] (Table 1, Table 1 (suite). There was no publication bias in the assessment of each glomerular disease.

**Table 1:** summary of extracted data from all included studies

Last name of first author	Study year	Country of study	African sub-region	Mean age (years)	Sample size	Male (%)	Population studied (A/C/A+C)
Ayach *et al*.	2011	Morocco	North Africa	19	77	61	A + C
Ali *et al*.	2018	Sudan	North Africa	5.2	330	66.7	C
Aatif *et al*.	2012	Morocco	North Africa	40.4	171	62.7	A
Abdel-Hafiz *et al*.	2017	Egypt	North Africa	10.51	221	46.19	C
Abumregha *et al*.	2020	South Africa	Southern Africa	6.2	231	52.4	C
EL-Hassan *et al*.	2016	Sudan	North Africa	33.6	100	42	A
Ali *et al*.	2017	Sudan	North Africa	7.7	130	60	C
Faye *et al*.	2016	Senegal	West Africa	29.7	156	72	A
N'dah *et al*.	2022	Cote d'voire	West Africa	33.83	153	52.9	A
Muthui *et al*.	2010	Kenya	East Africa	27.2	193	53.37	A
Nadium *et al*.	2013	Sudan	North Africa	34.6	83	54.9	A
Okpechi *et al*.	2011	South Africa	Southern Africa	36.8	1284	45.2	A
Souilmi *et al*.	2015	Morocco	North Africa	10.05	112	51.79	C
Lemrabott *et al*.	2020	Senegal	West Africa	33.8	1154	53.5	A
Vester *et al*.	2022	Gambia	West Africa	14.9	121	58.7	A+C
Vermeulen *et al*.	2019	South Africa	Southern Africa	33.5	1848	50	A
Zajjari *et al*.	2015	Morocco	North Africa	44.82	130	66.2	A

Abbreviations: A=Adults; C=Children; AUA: asymptomatic urinary abnormalities; IHC: immunohistochemistry; IF: immunofluorescence; EM: electron microscopy; GN: glomerulonephritis

**Table 1(suite):** summary of extracted data from all included studies

Last name of first author	Nephrotic syndrome (%)	Nephritic syndrome (%)	AUA (%)	Haematuria (%)	Abnormal kidney function (%)	Proportion of primary GN (%)	Histopathologic Assessment	Quality
Ayach *et al*	100	0	0	0	0	97.4	LM, IF	Medium
Ali *et al*	100	0	0	19.1	7.3	100	LM	Medium
Aatif *et al*	60.3	1.9	6.2	0	31.6	52	LM, IF	High
Abdel-Hafiz *et al*	43.89	6.79	0	10.41	15.38	63.35	LM, IF, EM	High
Abumregha *et al*	100	0	0	0	11.2	100	LM, IF, EM	High
EL-Hassan *et al*	0	100	0	0	0	59	LM, IF	Medium
Ali *et al*	100	0	0	57	15	100	-	Medium
Faye *et al*	100	0	0	0	0	100	-	Medium
N'dah *et al*	49	49	0	0	35.3	83.72	LM, IF	High
Muthui *et al*	76.17	6.74	0	0	16.6	89.6	LM	High
Nadium *et al*	46.5	7	8.5	0	0	85.5	LM, IF	High
Okpechi *et al*	52.5	5.8	13.6	0.3	6.4	34.97	LM, IF, EM	High
Souilmi *et al*	25.9	1	21.4	3.6	13.4	71.42	LM, IF	High
Lemrabott *et al*	78.3	0	0	0	0		-	Medium
Vester *et al*	77	0	0	78	28	95.9	-	High
Vermeulen *et al*	47.7	0	8.1	0	0	39.7	LM, IF, EM	High
Zajjari *et al*	61.5	2.3	5.4	0	30.8	46.2	LM, IF	High

Abbreviations: A=Adults; C=Children; AUA: asymptomatic urinary abnormalities; IHC: immunohistochemistry; IF: immunofluorescence; EM: electron microscopy; GN: glomerulonephritis

**Figure 2:**
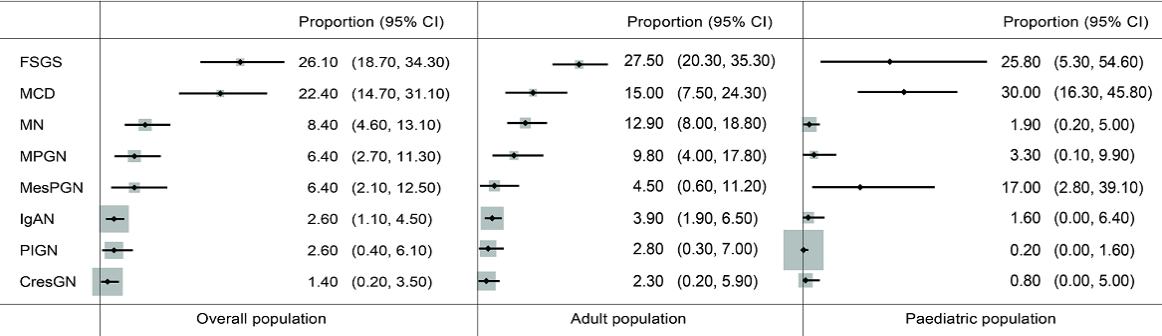
pooled prevalence of primary glomerular diseases in adult and paediatric populations in Africa

**Figure 3:**
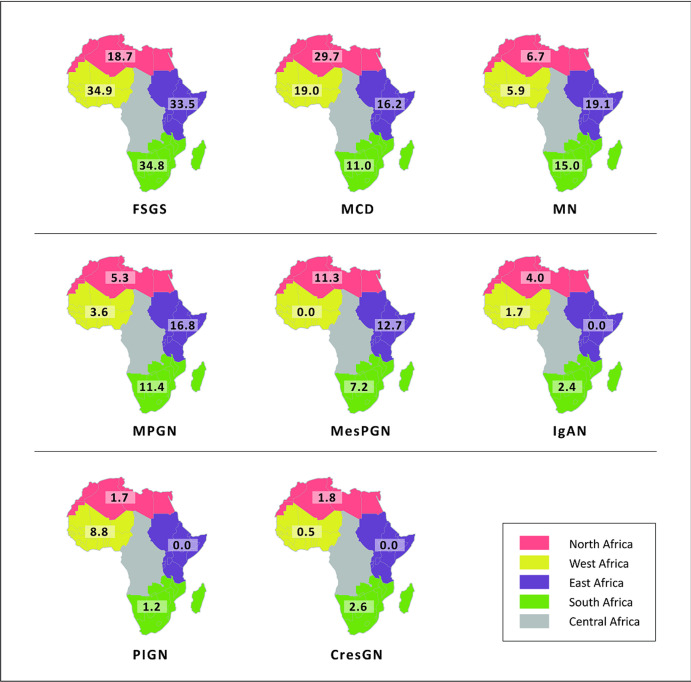
prevalence of primary glomerular disease sun-types across African sub-regions; FSGS-focal segmental glomerulosclerosis; MCD-minimal change disease; MN-membranous nephropathy; MPGN-membranoproliferative glomerulonephritis; MesPGN-mesangioproliferative glomerulo nephritis; IgAN - IgA nephropathy; PIGN - post-infectious glomerulonephritis; CresGN - crescentic glomerulonephritis

**Minimal change disease (MCD):** minimal change disease accounted for 22.4% (95% CI 14.7-31.1%, I^2^=96.8%, p<0.001 for heterogeneity) of all primary glomerular disease subtypes with non-significant variation across the African regions (Figure 2, Annex 2). In North Africa, MCD was seen in 29.7% (95% CI: 15.0-46.8%); West Africa in 19.0% (95% CI: 5.2-38.4%); Southern Africa in 11.0% (95% CI: 5.8 - 17.6%), and East Africa in 16.2% (95% CI: 11.0 - 22.1%); p=0.12). However, the prevalence of MCD was higher in paediatric studies than in studies focused on adults alone (30.0% [95% 16.3-45.8%] versus 15.0% [7.5-24.3%], p=0.08) (Figure 2, Figure 3).

**Focal segmental glomerulosclerosis (FSGS):** the overall pooled prevalence of FSGS was 26.1% (95% CI 18.7-34.3%, I^2^=96.1%, p<0.001 for heterogeneity); this prevalence was higher in studies of adults than those of children (27.5% [95% CI: 20.3-35.3%] versus 25.8% [95% CI: 5.3-54.6%]; p=0.80) (Figure 2). The prevalence was similar across sub-Saharan Africa regions: West Africa (34.9 [95% CI: 27.8 - 42.3%]), East Africa (33.5 [95% CI: 26.7 - 34.3%]), and Southern Africa (34.8 [95 CI: 9.5 - 66.1%]) and was significantly lower in North Africa (18.7% [95% CI: 10.4 - 28.7%]; p<0.001) (Figure 3, Annex 3).

**Membranous nephropathy (MN):** the pooled proportion of membranous nephropathy (MN) was 8.4% (95% CI 4.6 - 13.1%); I^2^=94.5%, p<0.001 for heterogeneity. There was a significantly higher proportion of MN in adults compared to children [12.9% (95% CI 8.0-18.8) versus 1.9% (95% CI 0.2-5.0%), p<0.001 (Figure 2). Pooled studies by sub-regions showed that East Africa has the highest prevalence (19.1% [95% CI: 13.5 - 25.3%]) while West Africa has the lowest prevalence (5.9% [95% CI: 1.6 - 12.5%]); p=0.007 (Figure 3, Annex 4).

**Membranoproliferative glomerulonephritis (MPGN):** the pooled prevalence of MPGN was 6.4% (95% CI 2.7-11.3%, I^2^=95.8%, p<0.001). Studies involving adult patients had a higher prevalence than those involving only paediatric age groups (9.8% [95% CI 4.0-17.8%] versus 3.3% [95% CI: 0.1-9.9%]; p=0.084) (Figure 2). Across regions, MPGN prevalence was significantly higher in East Africa (16.8%) than in other regions: Southern Africa (11.4%), North Africa (5.3%), and West Africa (3.6%); p=0.006 (Figure 3, Annex 5).

**Non-IgA mesangioproliferative glomerulonephritis (MesPGN):** mesangioproliferative glomerulonephritis had a pooled proportion of 6.4% (95% CI: 2.1-12.5%, I^2^=97.2%, p<0.001) with a significantly higher prevalence in children compared to adults [17.0% (95% CI: 2.8-39.1%) versus 4.5% (95% CI: 0.6-11.2%); p=0.002) (Figure 2). MesPGN occurred most frequently in North Africa (11.3% [95% CI: 2.5 - 24.7%]) than in other African sub-regions (Figure 3, Annex 6).

**IgA nephropathy (IgAN):** the pooled prevalence of IgAN was 2.6% (95% CI 1.1-4.5%, I^2^=86.7%, p<0.001) with a significantly higher frequency in the adult than in paediatric populations (3.9% [95% CI 1.9-6.5%] versus 1.6% [95% CI: 0.0-6.4%]; p=0.003) (Figure 2). IgA nephropathy occurred at a significantly higher frequency in North Africa (4.0% [95% CI: 1.1 - 8.3%]); p=0.02, compared to other regions (Figure 3, Annex 7).

**Post-infectious glomerulonephritis (PIGN):** post-infectious glomerulonephritis had a pooled proportion of 2.6% (95% CI: 0.4-6.1%, I2=95.6%, p<0.001) with no significant difference in prevalence between adults and children (2.8% [95% CI: 0.3-7.0% versus 0.2% [95% CI: 0.0-1.6%]; p=0.15) (Figure 2). A low pooled prevalence was observed across regions with no significant difference (p=0.12) (Figure 3, Annex 8).

**Primary crescentic glomerulonephritis (CresGN):** primary CresGN was the least frequent, with a pooled prevalence of 1.4% (95% CI: 0.2-3.5%, I2=92.3%, p<0.001) and no significant difference in frequency between adults and paediatric populations (p=0.13) (Figure 2). The frequency was highest in Southern Africa (2.6% [95% CI: 0.0 - 11.0%]) with no significant difference across regions (p=0.20) (Figure 3, Annex 9).

## Discussion

This study identifies the burden of primary glomerular diseases in Africa and highlights important demographic and sub-regional differences in the occurrence of glomerular disease sub-types. Although not a direct extension of a previous systematic review [19], shows that there have been marked differences in the occurrence of primary glomerular diseases in the continent. The main finding from this study is the higher occurrence of FSGS and MCD across regions and age groups than other primary glomerular diseases. Other important findings include the low frequency of IgAN and PIGN and the low number of biopsies reported from studies (average number of biopsies: 382 per study or 541.2 per year studied).

Glomerulonephritis is a common cause of kidney failure in most parts of sub-Saharan Africa [5,6]. Most primary glomerular diseases have a peak incidence in younger and middle-aged people and may account for the younger average age of patients with kidney failure reported in Africa, often being less than 50 years [9-12,45,46]. Kidney failure is associated with a high rate of premature mortality in Africans, often due to unavailable therapeutic measures or a high cost of treatment [46,47]. Early disease detection and instituting measures that slow or halt disease progression still remain the best options for reducing the burden of kidney failure and poor outcomes in Africa [46-48].

Evidently, the high FSGS occurrence in sub-Saharan Africa may be related to the high prevalence of apolipoprotein L1 (APOL1) kidney risk variants in these regions [49-51]. Low prevalence of APOL1 in Arabs [52] and North Africans [53] may also explain the lower prevalence of FSGS observed in North Africa. Furthermore, the high prevalence of various environmental factors such as sub-clinical viral infections (e.g. parvovirus B19 [54] cytomegalovirus, Epstein-Barr Virus, and Simian virus [55]), which have been shown to be associated with FSGS lesions may also have a role in the observed prevalence. In most countries in Africa, kidney biopsies are often evaluated only using light microscopy and without immunohistochemistry or electron microscopy, which improves diagnostic accuracy and aids in differentiating primary from secondary patterns [56]. It is unclear if this contributed to the high prevalence of FSGS, given that several advanced glomerular lesions may be seen with sclerosis.

Although some studies have identified FSGS to be more common than MCD in children [57,58] our study showed that MCD is more common in children than adults (Figure 2). Moreover, a recent systematic review of paediatric studies of patients with nephrotic syndrome in Africa over half a century showed MCD to have a higher prevalence than FSGS (38% [95% CI: 36-40%] versus 24% [95% CI: 22-25%]) [59]. However, even though this study shows a higher frequency of MCD in children, the ratio of MCD to FSGS in children is much lower than that in a previous study [19] and could suggest a higher occurrence of FSGS than MCD in African children. A study from South Africa showed that FSGS, which was once considered uncommon in children, has recently emerged as one of the most challenging forms of nephrotic syndrome across all racial groups, particularly in Black children [58]. Studies from other African countries are needed to corroborate this, as it shows that childhood nephrotic syndrome cannot always be assumed to be due to MCD [20], especially if it is steroid resistant. Guidelines for the treatment of steroid-resistant nephrotic syndrome in children identify FSGS as the predominant type and strongly recommend early kidney biopsy and genetic testing (where available) [60]. The reduced proportion of cases of PIGN in adults and children may be related to improving health services, widespread availability of antibiotics, and changes in the organisms causing PIGN. Due to these factors, epidemics of PIGN still occur, albeit in reduced frequencies [61], and pockets of African communities still have a high prevalence of PIGN [44].

The pooled prevalence of primary MN was 1.9% in children compared to 12.9% in adults. Primary MN in Africa is also a disease of adults like in other climes [62]. The higher prevalence of MN in Southern Africa may be related to the greater numbers of individuals with Caucasian heritage in South Africa. Another reason for the low MN frequency in West Africa may be the decreasing number of kidney biopsies and the unavailability of serum and tissue-based tests for Phospholipase A2 receptor 1 (PLA2R) and thrombospondin type-1 domain containing 7A (THSD7A). Despite being the commonest primary glomerulopathy globally, IgAN is still rare among black Africans [63]. Studies from North Africa reported a pooled prevalence close to that obtained in Europe but lower than the prevalence reported among Asian populations [64]. Among various factors, the hygiene hypothesis, an alteration in the immune balance of the T helper 1 and T helper 2 subsets, has been proposed as a mechanism to explain differences in glomerular disease frequencies, particularly of IgAN, across countries [65]. Genetic factors may also account for the low frequency in Africans [66].

It is also possible that the aforementioned factors contribute to major differences in the frequencies of primary GNs reported in this study in comparison with other world regions (Table 2) [18]. In an international kidney biopsy survey involving 29 centers across 18 countries, FSGS was identified to be the most common primary GN in North America (19.1%) and Latin America (15.8%), while IgAN was the most common in Asia (39.5%) and Europe (22.1%) [18]. Despite these differences, the reporting of FSGS and MCD from Africa appears to be much higher than reported from other world regions. Whether this represents the true frequency of these glomerular diseases or reporting due to a lack of facilities to adequately distinguish other glomerular conditions is currently unknown. However, there is a widespread lack of facilities for immunofluorescence and electron microscopy in sub-Saharan Africa, and the practice of kidney biopsy is still not well-developed among renal care practitioners in many countries in sub-Saharan Africa [19,56,67]. A recent study that assessed challenges in the diagnosis and management of glomerular diseases in resource-limited settings (mainly Africa and Asia) reported major system-level barriers that impede the implementation of guideline-driven approaches for the diagnosis and treatment of patients with glomerular disease [68]. These barriers included a low performance of kidney biopsies, cost of diagnostic work-up (e.g. serologic testing) and care, and low utilization of diagnostic techniques (LM, IHC/IF, and EM are used in diagnostic work-up of only 29.2% of biopsies in Africa) [68]. This could suggest over-diagnosis of FSGS and MCD from several of the studies included in our study, given that only 23.5% used LM, IHC/IF, and EM for reporting the biopsies. Only studies from North Africa and Southern Africa used all three methods of assessment to evaluate histologies. Given the importance of glomerular diseases in the overall burden of CKD and kidney failure in Africa, there is a need to improve infrastructure to improve diagnostic capacity.

**Table 2:** frequencies of primary glomerular disease prevalence across world regions

	North America (USA/Canada)* (n=23,391)	Europe* (n=15,042)	Asia* (n=1,609)	Latin America* (n=2,561)	Africa (this study) (n=6,494)
**FSGS**	19.1	14.9	6.9	15.8	26.1
**MCD**	4.1	6.4	3.4	6.8	22.4
**IgAN**	11.8	22.1	39.5	6.1	2.6
**MN**	11.6	12.5	10.1	11.1	8.4
**MPGN**	2.6	3.7	1.1	2.8	6.4
**MesPGN**	1.9	1.8	0.3	2.4	6.4
**PIGN**	0.8	0.8	1.6	1.7	2.6

* Data from O'Shaughnessy *et al*. Nephrol Dial Transplant. 2018 Apr 1;33(4):661-669. doi: 10.1093/ndt/gfx189. Abbreviations: FSGS: focal segmental lomerulosclerosis; MCD: minimal Change Disease; IgAN: IgA Nephropathy; MN: membranous nephropathy; MPGN: membranoproliferative GN; MesPGN: mesangioproliferative GN (non-IgA); PIGN: post-infectious GN

A limitation of this study is the lack of studies from Central Africa which highlights the lack of kidney biopsies across several African countries and leaves an information gap in the study. It was also not possible for us to assess how the primary disease was ascertained, given that serological markers to distinguish these from secondary glomerular diseases were not reported. However, this was not a limitation of our study given that the studies included did not report these. Finally, it would have been beneficial to conduct an analysis showing the temporal trends of the different histologic sub-types in Africa, but this study covered a relatively short period (12 years) for such changes to occur. Despite these perceived limitations, our study has important values to guide decision-making regarding the occurrence of common glomerular diseases and the need for improving services to aid diagnosis.

## Conclusion

Primary glomerular diseases remain a leading cause of kidney failure in Africa and affect a relatively younger population than in other climes. Overall, FSGS is the most common histologic subtype of primary glomerular diseases in Africa in adult and paediatric populations, followed by MCD. It is important to understand how high the prevalence of FSGS and MCD affects the overall kidney disease burden and outcomes in the region. Efforts to improve kidney biopsy practice as a means of early disease detection are needed in Africa, given the contribution of glomerular diseases to the burden of kidney failure on the continent. This will include increasing the infrastructure and workforce (nephropathologists, and pathology technicians) required for such services.

### What is known about this topic

- *Primary glomerular diseases are an important cause of kidney failure in Africa;*
- *For the above reason, kidney failure occurs at a relatively younger age at diagnosis, increasing the burden on an already dysfunctional health system*.

### What this study adds

- *The most common primary glomerulopathy among adults in Africa is FSGS with a pooled estimate of 27.5% (95% CI 20.3 - 35.3%);*
- *The most common primary glomerulopathy among children in Africa is FSGS with a pooled estimate of 25.8% (95% CI 5.3 - 54.6%);*
- *Overall, FSGS is the most common (26.1%) primary glomerulopathy in Africa, followed in descending order by minimal change disease (22.4%), membranous nephropathy (8.4%), membranoproliferative glomerulo nephritis (6.4%), mesangioproliferative glomerulonephritis (6.4%), IgA nephropathy (2.6%), post-infectious glomerulonephritis (2.6%) and crescentic glomerulonephritis (1.4%)*.

## Annexes

**Annex 1**: search strategy (PDF-298KB)

**Annex 2**: pooled prevalence of minimal change disease by region (PDF-288KB)

**Annex 3**: pooled prevalence of focal segmental glomerulosclerosis by region (PDF-271KB)

**Annex 4**: pooled prevalence of membranous nephropathy by region (PDF-626KB)

**Annex 5**: pooled prevalence of membrano proliferative glomerulonephritis by region (PDF-296KB)

**Annex 6**: pooled prevalence of non-IgA mesangial proliferative glomerulonephritis by region (PDF-291KB)

**Annex 7**: pooled prevalence of IgA nephropathy by region (PDF-291KB)

**Annex 8**: pooled prevalence of post-infectious glomerulonephritis by region (PDF-289KB)

**Annex 9**: pooled prevalence of primary crescentic glomerulonephritis by region (PDF-290KB)
